# Age-related macular degeneration: a review of current therapies and new treatments

**DOI:** 10.5935/0004-2749.20200082

**Published:** 2024-02-11

**Authors:** Vinicius Kniggendorf, Juliana L. Dreyfuss, Caio V. Regatieri

**Affiliations:** 1 Department of Ophthalmology and Visual Sciences, Escola Paulista de Medicina, Universidade Federal de São Paulo, São Paulo, SP, Brazil; 2 Department of Biochemistry, Molecular Biology Division, Escola Paulista de Medicina, Universidade Federal de São Paulo, São Paulo, SP, Brazil

**Keywords:** Macular degeneration, Angiogenesis inhibitors, Drug therapy, Choroidal neovascularization, Vascular endothelial growth factor A, Degeneração macular, Inibidores da angiogenese, Tratamento farmcalológico, Neovascularização de coroide, Fator A de crescimento do endotélio vascular

## Abstract

Age-related macular degeneration is the leading cause of vision loss in elderly individuals, as well as a medical and socio-economic challenge. The treatment of dry age-related macular degeneration is based on vitamin supplementation. New treatment studies are focused on preventing the progression of degeneration and repopulating the atrophic macula. Recently, research on the treatment of neovascular age-related macular degeneration experienced a breakthrough with the advent of anti-vascular endothelial growth factor inhibitors. Nevertheless, despite the fact that ranibizumab, aflibercept, and bevacizumab are effective in reducing severe visual impairment, patients usually lose some vision over time. Therefore, the search for new therapies and diagnostic methods is fundamentally important. Current studies are focused on new anti-vascular endothelial growth factor drugs, nucleoside reverse transcriptase inhibitors, antibody against sphingosine-1-phosphate, anti-platelet-derived growth factor, gene therapy, and RNA interference. The results of ongoing clinical studies may improve the therapy of age-related macular degeneration.

## Abstract (pt)

Degeneração macular relacionada à idade (DMRI) é a principal causa de perda de visão em pessoas idosas. É também um desafio médico e socioeconômico. O tratamento da degeneração macular relacionada à idade seca baseia-se na suplementação vitamínica. Novos tratamentos estão focados na prevenção da progressão da degeneração e tentativas de repovoar a mácula atrófica. A degeneração macular relacionada à idade neovascular experimentou um grande avanço com o advento dos inibidores do fator de crescimento endotelial anti-vascular (anti-VEGF); no entanto, apesar do ranibizumab, aflibercept e bevacizumab serem eficazes na redução do comprometimento visual grave, os pacientes geralmente per dem visão ao longo do tempo. Portanto, a busca por novas terapias, tratamentos e diagnósticos é de fundamental importância. Os estudos estão focados em novos fármacos sobre fator de crescimento endotelial anti-vascular, inibidores nucleosideos da transcriptase reversa, anticorpos contra esfingosina-1-fosfato, fator de crescimento derivado de plaquetas, terapia genética e RNA de interferência. A terapia para degeneração macular relacionada à idade está prestes a melhorar como resultado desses estudos clínicos em andamento.

## AGE-RELATED MACULAR DEGENERATION (AMD)

AMD is the leading cause of vision loss in elderly individuals (>50 years old) in industrialized countries, affecting 10%-13% of adults aged >65 years in North America, Europe, Australia and, most recently, in Asia^(1^,^2)^.

AMD is a medical and socio-economic challenge because it has a similar impact on patient quality of life to that reported for acquired immunodeficiency syndrome, kidney failure, and stroke^(3^,^4)^. Owing to the increased life expectancy and exposure to environmental risk factors (e.g., atherosclerosis, obesity, and smoking), the incidence of AMD is expected to increase^(4^,^5)^.

Studies confirmed the strong relationship between age and AMD, probably as a result of the complex interaction of metabolic, functional, genetic, and environmental factors. This multifactorial process causes im portant changes in the macular structure (i.e., choriocapillary, Bruch’s membrane, retinal pigmented epi thelium, and photoreceptors) that define the manifestations of the disease^(6)^.

With aging, focal deposition of acellular debris occurs between the retinal pigment epithelium and Bruch’s membrane. On fundus examination, these focal deposits (termed drusen) appear as round yellow-white punctate accumulations under the retinal pigment epithelium (RPE) in the macula or retinal periphery. Drusen can be classified as small (diameter: <63 µm), medium (63-124 µm), or large (>124 µm)^(7)^.

## AMD CLASSIFICATION

There are two clinical forms of AMD, commonly referred to as non-neovascular (dry AMD) and neovascular (wet AMD). The classification proposed by the Age-Related Eye Disease Study (AREDS) is currently the most commonly used. Early AMD is characterized by the presence of a few (<20) small drusen or retinal pigmentary changes. Intermediate AMD is defined as the presence of at least one large drusen, numerous medium-sized drusen, or geographic atrophy that does not extend to the center of the macula. Advanced or late AMD is classified as either the atrophic form (i.e., geographic atrophy that extends to the center of the macula) or the neovascular form (i.e., choroidal neovascularization and its sequelae)^(7^,^8)^.

Clinically, choroidal neovascularization is characterized by fibrovascular tissue, subor intra-retinal hemorrhage and fluids, lipids, and possibly RPE atrophy. The new vessels grow from the choriocapillary layer, passing through gaps through digestion of Bruch’s membrane, invading the space below the RPE, and eventually the subretinal space. This process leads to disruption of Bruch’s membrane, the RPE, and photoreceptors^(8^,^9)^.

### Angiogenesis and neovascular AMD

Angiogenesis is the formation of new blood vessels from pre-existing vasculature. The process is regulated by several factors capable of stimulating or inhibiting the process of vessel formation. Blood vessels are composed of endothelial cells that form the internal wall of the vessel and perivascular cells (referred to as pericytes), and vascular smooth muscle cells that surround the surface of the vascular tube^(10)^.

The stages of angiogenesis include: 1) stimulation of en dothelial cells by angiogenic factors (e.g., hypoxiain duci ble factor 1 [HIF-1], vascular endothelial growth factor (VEGF), and fibroblast growth factor 2 [FGF-2]); 2) degradation of the basement membrane surrounding the vessel; 3) proliferation and migration of endothelial cells to the site of vessel formation; 4) maturation of new vessels by secretion of growth factors (e.g., angiopoietin and platelet-derived growth factor [PDGF]) that attract support cells, such as pericytes and smooth muscle cells; and 5) formation of the basement membrane^(11^,^12)^.

The pathogenesis of choroidal neovascularization is not fully understood, although there have been descriptions of diverse mechanisms. An imbalance between pro-angiogenic factors (e.g., VEGF) and anti-angiogenic factors (e.g., pigment epithelium-derived factor) is necessary for the development of angiogenesis^(13)^. Pathological conditions (e.g., hypoxia, ischemia, or inflammation) influence the regulation of these growth factors and shift the balance in favor of pro-angiogenic molecules, such as VEGF.

Drusen deposits between Bruch’s membrane and the RPE may contain bioactive complement fragments (e.g., C3a and C5a) that induce the expression of growth factors and cytokines, stimulating the chemotaxis of inflammatory cells^(14^,^15)^. These findings, coupled with evidence of increased inflammatory biomarkers in individuals at risk of progression of AMD, strengthen the theory of age-related inflammatory disease. Furthermore, PDGF-B is involved in the development of retinal vasculopathies as well as the maturation of new vessels^(10^,^16)^. Hence, it appears to be a novel target molecule.

Therefore, the upregulation of growth factors expression (including VEGF, tumor necrosis factor-α, PDGF, and other cytokines), as well as modifications and degradation of the basement membrane may be a common pathway of inflammation and formation of new vessels in AMD.

## TREATMENT OF DRY AMD

Age, smoking, exposure to radiation, and genetic predisposition are risk factors for this disease^(8)^. Oxidative stress can be prevented by cessation of smoking, protection from ultraviolet A and B radiation using coated lenses, and intake of antioxidative molecules in the diet or supplementation^(17^-^19)^.

Studies investigating the treatment of dry AMD are focused on preventing the progression of degeneration or repopulating the atrophic macula.

The results of AREDS1 and AREDS2 did not demonstrate benefits from the combination of antioxidant vitamins in patients with early AMD, because they did not reduce the rate of progression to the intermediate stage. Nevertheless, patients with intermediate AMD and advanced AMD (in one eye) benefited from vitamin supplementation, reducing progression of the disease in 25% of patients and the risk of losing vision within 5 years (≥3 lines) by 19%. The investigators of AREDS2 recommended modifying the original AREDS1 formula, with the replacement of beta-carotene by lutein and zeaxanthin because of its association with lung cancer. They also recommended supplementation with daily doses of vitamin C (500 mg), vitamin E (400 UI), zinc (80 mg), copper (2 mg), lutein (10 mg), and zeaxanthin (2 mg) for intermediate AMD and advanced AMD in one eye^(20^,^21)^.

Despite the findings reported by the AREDS studies, the use of vitamins and minerals remains controversial. A recent systematic review involving 76,756 subjects questioned the value of vitamin and mineral supplementation. The conclusion drawn was that more evidence of benefit is required prior to recommending such supplementation, despite the fact that supplements are generally safe^(22)^.

Studies investigating genetic risk factors have identified some degeneration mechanisms (including complement factor H) as pathogenic pathways^(17)^. This allows the direction of research toward new treatment possibilities and therapeutic strategies that interfere with these pathways^(23)^. Preclinical studies investigating other complement factors (e.g., properdin, factor-B, C3, and C5) are currently in development^(24)^.

Pegcetacoplan (APL-2; Apellis Pharmaceuticals) binds to C3 and C3b and blocks complement activation. A phase II study (FILLY) compared intravitreal injection (monthly and bi-monthly) with a control group. The results were promising, with a reduction in the rate of geographic atrophy of 29%^(25)^. Although the monthly injection was associated with a better outcome (47% red uction), the risk of neovascular AMD was 18% compared with 8% for the bi-monthly injection and 1% in the control group^(26)^. A phase III trial is currently in the recruitment phase (NCT03525600).

Lampalizumab (Genentech/Roche) is a humanized monoclonal antibody that blocks complement factor D. The latter is involved in the activation of the alternative complement pathway, related to the development of AMD^(27)^. The results of a phase II trial suggested that lampalizumab reduces the rate of atrophy progression; however, the phase III trial did not show significant differences compared with the effects of sham treatments over 48 weeks of follow-up^(28^,^29)^.

Cell therapy is an alternative strategy in the absence of cells to be preserved using neuroprotective agents. There have been various *in vitro* and animal model trials of cell therapies, including stem cells (i.e., embryonic, cord blood, bone marrow, and RPE cells)^(30)^. Clinical trials for these therapies have recently been approved. Several clinical trials are underway to test the safety and efficacy of stem cell transplantation for the repair of the RPE in patients with advanced vision loss. Subretinal transplantation of human embryonic stem cell-derived RPE - MA09-hRPE in patients with Stargardt’s macular dystrophy or dry AMD with geographic atrophy is the subject of one of the most advanced studies^(31)^. Identification of the optimal delivery technique remains under investigation. Currently, the subretinal approach is being evaluated, thus far appearing to be the most efficient route. Nevertheless, future studies will investigate other possibilities, including substrates upon which the cells can be layered^(32)^. Induced pluripotent stem cells are also being studied as a source for transplantation; in this model, cells are modified to express the characteristics and functions of photoreceptors^(33)^.

## TREATMENT OF NEOVASCULAR AMD

Recently, research on the treatment of neovascular AMD experienced a breakthrough with the advent of anti-VEGF inhibitors that stabilize and improve visual acuity^(34)^. Studies showed that monthly injections of ranibizumab reduced the number of incident blindness cases from 16,268 to only 4,484 individuals in the USA and Australia, leading to a 72% reduction in the incidence of blindness over a 2-year period^(35^,^36)^.

VEGF is a pro-angiogenic growth factor that stimulates the proliferation and survival of vascular endothelial cells. VEGF also induces vessel permeability and plays an important role in the development and persistence of subretinal neovascular membranes in AMD^(37)^. The currently available pharmacotherapeutic agents for the treatment of exudative AMD block the biological effects of VEGF on the neovascular endothelium by preventing the binding of VEGF to its endothelial cell receptor. There are several anti-VEGF agents currently in clinical use, including ranibizumab, bevacizumab, and aflibercept.

### Current therapies

#### Ranibizumab

Ranibizumab (Lucentis, Genentech, Inc., San Francisco, CA, USA) was approved for use in June 2006. It is a recombinant fragment human immunoglobulin G1 (IgG1) kappa monoclonal antibody that binds to VEGF-A^(38)^.

The safety and efficacy of intraocular injections of ranibizumab have been tested in several clinical trials, including MARINA, ANCOR, FOCUS, PIER, and PrONTO^(39^-^43)^. Intravitreal injection of ranibizumab has been shown to be safe and effective in these clinical trials. Treatment with this drug leads to morphological improvements in the retina, including decreased area of choroidal neovascularization and decreased retinal thickness in patients with neovascular AMD. Compared with bevacizumab (a recombinant humanized IgG1 monoclonal anti-VEGF-A antibody), ranibizumab induced a greater reduction in central retinal thickness. However, this difference was not clinically significant, as visual acuity was comparable following the administration of the same treatment protocol^(44^-^46)^.

Another difference is that ranibizumab exhibits a higher affinity for VEGF than bevacizumab owing to its molecular characteristics^(47)^. Adverse events that may occur after intravitreal injection of ranibizumab are ocular inflammation and increased intraocular pressure. Endophthalmitis was observed in <1% of patients. Systemic effects, such as thromboembolic events, were reported only in patients treated with 0.5 mg of ranibizumab and were not statistically significant^(48)^

#### Bevacizumab

Bevacizumab (Avastin; Genentech, Inc., San Francisco, CA, USA) is also a recombinant humanized IgG1 monoclonal antibody that binds to the active forms of VEGF-A. It differs from ranibizumab in that it is a complete IgG molecule weighing 149 kDa (ranibizumab is the antigen-binding fragment of the antibody). Bevacizumab binds to all VEGF isoforms, and was approved by the Food and Drug Administration (USA) for the treatment of metastatic colorectal cancer, breast cancer, and lung cancer. Bevacizumab was initially used systemically for the treatment of exudative AMD in the SANA cli nical trial, with all patients experiencing increased visual acuity and decreased central retinal thickness. The use of bevacizumab in AMD was reported for the first time in 2006^(49)^.

Despite the fact that several studies have demonstrated the safety and efficacy of intravitreal injection of bevacizumab for the treatment of neovascular AMD and diabetic retinopathy, the drug has not been approved for intraocular use^(45^,^50)^. As it was only approved for the treatment of some types of cancer, the pharmaceutical preparation requires fractionation for intravitreal use. This is because the required dose is markedly lower than the systemic dose, and concerns related to the drug fractionation are important for avoiding contamination and endophthalmitis^(51)^.

#### Aflibercept

Aflibercept (EYLIA; Regeneron Pharmaceuticals, Inc., Tarrytown, NY, USA and Bayer Healthcare Pharmaceuticals, Berlin, Germany) has recently been approved for the treatment of neovascular AMD^(2)^. Aflibercept, or VEGF Trap-Eye, is a protein composed of extracellular segments of VEGF1 (VEGF receptor 1 [VEGFR1]) and VEGF2 (VEGFR2) receptor domains fused with the Fc portion of human IgG1, thereby conferring anti-angiogenic potential to the drug^(52)^. Aflibercept functions as a soluble receptor for VEGF, binding strongly to VEGF, consequently preventing VEGF from binding to cellular receptors. Prevention of the binding of VEGF to the VEGFRs of cells inhibits angiogenesis^(53^,^54)^.

The clinical trials VIEW 1 and VIEW 2 investigated the safety and efficacy of VEGF Trap-Eye in neovascular AMD. The results in patients receiving intravitreal aflibercept were clinically equivalent to those of patients receiving monthly ranibizumab for maintenance of visual acuity. This result was also confirmed following the administration of aflibercept every 2 months, allowing for substantially reduced monitoring frequency and treatment. This evidence introduces a new treatment strategy for the management of neovascular AMD^(55)^.

#### Corticosteroids

Dexamethasone intravitreal implants and triamcinolone are corticosteroids that may also be used as adjuncts for treatment^(56)^. The LuceDex clinical trial was a pilot study, which showed that the combination of intravitreal dexamethasone and ranibizumab may be beneficial in the treatment of neovascular AMD^(57)^. The intravitreal dexamethasone implant Ozurdex (Allergan, Irvine, CA, USA), in combination with ranibizumab, was evaluated for the treatment of refractory neovascular AMD. The combined treatment was effective in stabilizing visual acuity, decreasing subretinal fluid, and improving central retinal thickness^(58)^.

## NEW THERAPIES FOR NEOVASCULAR AMD

Despite the fact that ranibizumab, aflibercept, and bevacizumab are effective in reducing severe visual impairment, patients with neovascular AMD usually lose some vision over time, and often lose the ability to read, drive, or perform other important daily activities^(59)^.

The most commonly used treatment regimens are monthly injections, *pro re nata* (PRN), and treat-and-extend. Monthly injections yield the best results in terms of visual acuity. However, this treatment is linked to the burden of frequent visits to the clinic, as well as loss of productivity and time, which are important factors for patients and caregivers^(60)^. PRN was a strategy to reduce the number of injections; the procedure is performed only following the detection of signs of disease. Unfortunately, recurrence is common after this treatment, possibly leading to worse final visual acuity^(46)^. The treat-and-extend regimen aims to reduce the number of injections, avoiding disease activity and recurrence by creating an individualized interval for the patient^(61)^. All available regimen are associated with treatment burden due to the half-life of the drugs and the requirement for frequent retreatments.

Therefore, the search for new therapies and diagnostic methods for neovascular AMD is of fundamental importance. Some medications and their characteristics are listed below.

### Phase I and II Studies

#### Squalamine lactate

Squalamine lactate is an amino sterol derived from the cartilage of the dogfish shark; it has been shown to possess anti-angiogenic properties. The intracellular me chanism of action is inhibition of several growth factors (e.g., VEGF, PDGF, and basic fibroblast growth factor) via blockade of cell membrane ion transporters^(62^,^63)^. In animal models of choroidal neovascularization, intraperitoneal injection with squalamine reduced neovascular membranes^(64)^. The MAKO study (Ohr Pharmaceutical, Inc.) was designed to evaluate the safety and efficacy of topical squalamine in combination with monthly intravitreal Lucentis. However, after 9 months, the investigators did not observe benefits. More studies are warranted to determine the efficacy and safety of this drug.

#### Sphingomab

Sphingomab™ (Lpath) is a monoclonal antibody against sphingosine-1-phosphate (S1P), which induces significant capillary formation. It acts as a pro-angiogenic factor that directly interacts with other growth factors, including the epidermal growth factor and VEGF2 receptors^(65)^. The effects of S1P on cell migration, proliferation, and protection from cell death suggest that Sphingomab may be an effective therapeutic approach for wet AMD^(66)^.

#### Inhibition of nucleoside reverse transcriptase

Nucleoside reverse transcriptase inhibitors (NRTIs) are a class of drugs used for the treatment of acquired immunodeficiency syndrome, that may also be effective in treating AMD. Reports demonstrated that NRTIs blocked AMD in mice and were effective in experiments involving cultured human retinal cells. It was hypothesized that NRTIs halt reverse transcription and prevent RPE cell death in dry AMD; they also block the inflammasome, a complex of proteins that promote inflammation. Therefore, NRTIs may also exert effects on wet AMD^(67)^.

#### Gene therapy

AVA-101 (Avalanche Biotechnologies) is a gene therapy comprised of the adeno-associated virus 2 (AAV2) vector with a gene encoding soluble fms-like tyrosine kinase-1 (sFlt-1), a natural anti-VEGF protein. A phase IIa clinical study demonstrated that this therapy was well tolerated with a favorable safety profile in patients with wet AMD. The results also showed an improvement in best-corrected visual acuity. AVA-101 is being developed as a subretinal gene therapy injection to provide a safe and effective treatment for wet AMD that is durable and reduces the requirement for frequent anti-VEGF injections^(68^,^69)^. Adverum Biotechnologies (Menlo Park, CA, USA) and Sanofi Genzyme (Framingham, MA, USA) have completed phase I/II studies of AAV vectors expressing sFlt-1, which acts as a VEGF-trap. Both studies demonstrated the safety of AAV2-sFlt delivered either intravitreally or subretinally; however, the data showed limited efficacy^(70)^. Nevertheless, gene therapy is expected to become an important alternative for the treatment of AMD.

#### Inhibitors of tyrosine kinases

Vatalanib (Novartis, Basil, Switzerland) is an oral ty rosine kinase inhibitor that binds to the intracellular kinase-like domain of all VEGFR subtypes^(71)^. Another tyrosine kinase inhibitor, pazopanib (Glaxo Smith-Kline, Philadelphia, PA, USA), is a topical drug that selectively inhibits VEGFR 1, 2, and 3, the PDGF receptor (PDGF-R), and c-KIT^(72)^. A clinical trial did not demonstrate be nefits after treatment with pazopanib eye drops compared with the effects of intravitreal injections of ranibizumab alone^(73)^.

#### RNA interference

Bevasiranib (OPKO, Miami, FL, USA) is a small interfering RNA that induces gene silencing, designed to silence VEGF-A messenger RNA. Studies in animals demonstrated that intravitreal bevasiranib exhibited good biodistribution in the retina and RPE following delivery of the drug using a transfection agent. Drug delivery in humans remains a challenge, and the negative results observed in trials thus far may be explained by the naked small interfering RNA delivery system, which is different from the system used in animals. Bevasiranib is an interesting technology and a promising drug based on pharmaceuticals^(74^,^75)^.

#### Epiretinal radiotherapy

Radiation therapy is also being investigated for the treatment of neovascular AMD. Epiretinal application of strontium-90 alone or in combination with ranibizumab showed promising results^(76)^. A phase III clinical study (CABERNET) compared the combination of epimacular brachytherapy with ranibizumab versus ranibizumab alone. After 2 years of follow-up, the results did not demonstrate benefits in the combination group, suggesting that more studies are warranted. Radiation using a proton beam is another delivery method that is currently being studied for the treatment of neovascular AMD^(77)^.

### Novel treatments and Phase III studies

#### Ziv-aflibercept

Zaltrap (Sanofi-Aventis and Regeneron Pharmaceuticals) is a fully-humanized soluble recombinant fusion protein. It is similar to aflibercept, except for its excipients and higher osmolarity, and binds to all isoforms of VEGF-A, VEGF-B, and placental growth factor^(52^,^78)^. It was approved for the treatment of metastatic colorectal carcinoma and is currently under investigation for the treatment of wet AMD. Preliminary results demonstrated the safety and efficacy of intravitreal injections with ziv-aflibercept^(64^,^79^,^80)^.

#### Conbercept

Conbercept (KH902) is a VEGF trap-like molecule that blocks all isoforms of VEGF-A, VEGF-B, VEGF-C, and placental growth factor. It yielded favorable outcomes, with a possible longer duration versus other agents^(52^,^81^,^82)^. It was recently approved for the treatment of AMD in China. The phase III PHOENIX study demonstrated that intravitreal injections with conbercept (months 0, 1, 2, 5, 8, and 11) provided a mean gain of 10 letters and a statistically significant decrease in the central subfield thickness after 12 months of follow-up, demonstrating a promising therapeutic effect^(83)^. Compared with ranibizumab, conbercept exerted equivalent effects in terms of visual acuity. However, longer treatment intervals were observed, maintaining clinical benefits with a quarterly regimen^(83^,^84)^.

#### Pegpleranib

Pegpleranib (Fovista; Ophthotech Corp.) is an anti-PDGF agent administered in combination with anti-VEGF agents. Pegpleranib targets PDGF and blocks the interaction with its receptor. This leads to stripping or death of pericytes in the neovascular membrane, promoting a better response to anti-VEGF agents by reducing the protection of vessels^(85)^. The results of a phase II clinical trial demonstrated a 62% benefit versus anti-VEGF monotherapy for wet AMD, suggesting that pegpleranib may be a promising future treatment^(28)^. Nevertheless, the results of the phase III trial were disappointing. There was no significant difference in visual acuity with the combination of anti-VEGF agents and pegpleranib compared with anti-VEGF monotherapy^(86)^.

#### Faricimab

Faricimab (RG7716) (Roche, Switzerland), is an antibody that binds to angiopoietin 2 (Ang-2) and VEGF-A. Ang-2 leads to endothelial and vascular destabilization, breakdown of the blood-retinal barrier and inflammation. Simultaneous blockage of VEGF and Ang-2 may lead to improved efficacy and durability^(87)^. The phase I study demonstrated the safety and improvements in best-corrected visual acuity. The results of the phase II (STAIRWAY trial) results showed that 12- and 16-week regimens were comparable with monthly treatments with ranibizumab^(88^,^89)^. The phase III trials are currently in the recruitment stage (NCT03823287 and NCT03823300).

#### Abicipar pegol

Abicipar pegol (MPO112) is a VEGF-A antagonist belonging to the class of designed ankyrin repeat proteins that can be engineered to inhibit one or more target proteins^(90)^. It has a smaller molecular weight, higher target-binding affinity, and longer ocular half-life in aqueous humor (13 days vs. 7 days, respectively) compared with ranibizumab^(91^-^93)^. A phase II study demonstrated equivalence in terms of visual acuity gain versus ranibizumab^(94)^. Phase III clinical trials (SEQUOIA and CEDAR) were designed to compare 8- and 12-week intervals vs. monthly injections with ranibizumab; this may represent another breakthrough in anti-VEGF treatment for AMD.

#### Brolucizumab

Brolucizumab is a single-chain antibody fragment that inhibits VEGF-A; it is significantly smaller than other anti-VEGF agents and several times more concentrated^(95)^. Phase II and III studies comparing brolucizumab with aflibercept showed that visual acuity was comparable in both groups. More stable central subfield thickness, fewer unscheduled treatments, and higher rates of fluid resolution were observed in the brolucizumab group^(96^,^97)^. Phase III trials (HAWK and HARRIER) demonstrated that >50% of the patients were able to maintain a 12-week regimen over 48 weeks of follow-up, confirming that the half-life of brolucizumab is longer than that of aflibercept^(97)^. Brolucizumab appears to be an alternative treatment, offering favorable efficacy and injection frequency (12-week interval), and reduced monitoring burden.

**The Port Delivery System (PDS**)

The PDS is a novel drug delivery method designed to release ranibizumab by passive diffusion from a refilled implant, surgically-implanted in the pars plana. The phase II study Ladder Clinical Trial demonstrated its safety and tolerability. The PDS 100 mg/ml group showed visual outcomes comparable with those of monthly 0.5 mg ranibizumab; 79.8% of the patients did not require a refill for 6 months^(98)^. Therefore, PDS appears to be an option for reducing treatment burden; the phase III study is currently in the recruitment stage (NCT03677934).

## CONCLUSION

Several approaches have been used to inhibit choroidal neovascularization secondary to AMD. The current treatments effectively restrict the disease evolution, based on the inhibition of VEGF, one of the factors responsible for the proliferation of endothelial cells and the permeability of the neovascularization. The search for new diagnostic biomarkers and targets for the treatment of neovascularization secondary to AMD has been the subject of numerous studies worldwide, because this disease is the leading cause of blindness in adults aged >60 years.

The results of ongoing clinical studies may improve the therapy of AMD. New treatment options for dry AMD, slowing progression, or even reestablishing retinal cells, are becoming reality. For wet AMD, new drugs are being studied that will soon be approved and could lead to longer half-life in the vitreous, lower costs, and more potent anti-angiogenesis activity.
